# A Systemic Inflammation Response Score for Prognostic Prediction of Breast Cancer Patients Undergoing Surgery

**DOI:** 10.3390/jpm11050413

**Published:** 2021-05-14

**Authors:** Kaiming Zhang, Liqin Ping, Xueqi Ou, Meiheban Bazhabayi, Xiangsheng Xiao

**Affiliations:** 1 Department of Breast Oncology, Sun Yat-Sen University Cancer Center, State Key Laboratory of Oncology in South China, Collaborative Innovation Center for Cancer Medicine, Guangzhou 510060, China; zhangkm@sysucc.org.cn (K.Z.); ouxq@sysucc.org.cn (X.O.); hbbzby@sysucc.org.cn (M.B.); 2 Department of Medical Oncology, Sun Yat-Sen University Cancer Center, State Key Laboratory of Oncology in South China, Collaborative Innovation Center for Cancer Medicine, Guangzhou 510060, China; pinglq@sysucc.org.cn

**Keywords:** breast cancer, systemic inflammation response, preoperative, nomogram, prognosis

## Abstract

**Background:** Systemic inflammatory response is related to the occurrence, progression, and prognosis of cancers. In this research, a novel systemic inflammation response score (SIRS) was calculated, and its prognostic value for postoperative stage I-III breast cancer (BC) patients was analyzed. **Methods:** 1583 BC patients were included in this research. Patients were randomly divided into a training cohort (n = 1187) and validation cohort (n = 396). SIRS was established in the training cohort based on independent prognostic hematological indicator, its relationship between prognosis and clinical features was analyzed. Then, a nomogram consisted of SIRS and clinical features was established, its performance was examined by calibration plots and receiver operating characteristic curve analysis. **Results:** The SIRS was an independent prognostic indicator for BC patients, and a high-SIRS was related to multifocality, advanced N stage, and worse prognosis. Incorporating SIRS into a nomogram could accurately predict the prognosis of BC patients, the results of receiver operating characteristic (ROC) curve analysis showed that the area under the curve (AUC) of nomogram was up to 0.806 in training cohort and 0.905 in the validation cohort. **Conclusion:** SIRS was associated with the prognosis of patients with breast cancer. Nomogram based on SIRS can accurately predict the prognosis of breast cancer patients.

## 1. Introduction

Breast cancer is the most common newly diagnosed female cancer in China and worldwide. It is also the leading cause of cancer-related deaths in women [1]. American Joint Committee on Cancer (AJCC) stage is used for risk classification, treatment guidance and prognosis prediction in clinical practice [2]. However, breast cancer patients are quite heterogeneous, with even same AJCC-stage individuals treated with similar treatments obtaining significant heterogeneities of prognosis [3,4], suggesting that more accurate prognostic indicators should be incorporated into the overall prognostic analysis. With the development of molecular and precision medicine, several prognostic models of clinical variables combined with genotyping have been commonly used to predict the prognosis of preoperative breast cancer patients, such as 21-gene, MammaPrint, and PAM50 prognostic models [5,6,7]. However, their instability of testing, economic costs, and difficulty of implementation at the primary hospitals have limited the use of these models. It is necessary to establish a stable prognostic factor for breast cancer and construct a flexible prognostic model to perform better prediction of prognoses.

The immune status of breast cancer patients includes not only the local tumor immune microenvironment, but also systemic changes in immune status which could be indicated by hematological indicators [8]. Both systemic inflammation and local immune response play an important role in the progression of cancers [9]. Hematological indicators, which could reflect the immune status, are of great significance to the survival and prognosis of cancer patients. It has been reported that neutrophils and platelets are involved in the occurrence, proliferation and metastasis of tumor, and are related to the poor prognosis of cancer patients [10,11]. 

Several preoperative prognostic biomarkers based on circulating blood cells, such as lymphocyte-monocyte ratio (LMR), platelet-lymphocyte ratio (PLR), neutrophil-lymphocyte ratio (NLR), and serum albumin (ALB), which could indicate the host’s immune response to malignancy, have been developed to predict the prognosis of patients with various cancers [12,13]. In addition, inflammation inhibit albumin synthesis, and preoperative low serum ALB is significantly associated with reduced survival in patients with cancer [14]. The combined blood index of LMR and ALB can well predict the overall survival (OS) of patients with renal cancer [15]. It has also been suggested that LMR and NLR were independent prognostic factors for breast cancer [16]. 

Nevertheless, there are few reports about a comprehensive analysis of these hematological markers of inflammation. Therefore, the purpose of this research was to explore the prognostic value of systemic inflammation response score (SIRS) used as an independent factor to integrate all significant hematological indicators of inflammation in breast cancer patients. In addition, we attempted to develop and validate a nomogram for predicting OS in breast cancer patients based on the SIRS and other important clinical variables to provide clinicians with a more cost-effective, rapid, stable, and flexible prediction model.

## 2. Materials and Methods

### 2.1. Patients

We collected the information of consecutive patients diagnosed with invasive breast cancer between August 2012 to December 2015 in Sun Yat-sen University Cancer Center (SYSUCC). The inclusion criteria for patients were as follows: (1) female patients; (2) pathologically diagnosed with primary invasive breast cancer; (3) received modified radical mastectomy or breast conserving surgery. The exclusion criteria were: (1) incomplete laboratory data; (2) patients with distant metastasis (e.g., brain, liver, lung, bone); (3) received neoadjuvant therapy; (4) simultaneous presence of other primary cancers; (5) incomplete data such as multifocality, histological grade, T stage, N stage, human epidermal growth factor receptor 2 (HER2), progesterone receptor (PR), estrogen receptor (ER), and ki-67. All enrolled patients were randomly separated into a training or external validation cohort in 3:1 ratio. This study was approved by the ethics committee of the SYSUCC (identifier: 81372133).

### 2.2. Data Collection and Cut-off 

Hematology and laboratory parameters were obtained within three days before surgery. Age was defined as the age of the patient at the time of surgery. The pathology was reviewed by an experienced pathologist from SYSUCC. The size of the tumor was recorded as the longest diameter described in the pathology report. Multiple lesions were determined as more than or equal to two primary lesions. The number of lymph node metastases was determined by postoperative pathological examination, and the presence of distant metastases was determined by imaging findings. The classification of T stage and N stage were based on the diagnostic standard of the eighth edition of the American Joint Committee on Cancer (AJCC) criteria. The status of PR, ER, HER2 and ki-67 were determined by immunohistochemistry (IHC) or fluorescence in situ hybridization (FISH). The platelet-monocyte ratio (PMR), neutrophil-monocyte ratio (NMR), platelet-lymphocyte ratio (PLR), neutrophil-lymphocyte ratio (NLR) and lymphocyte-monocyte ratio (LMR) were calculated as follows: PMR = P/M; NMR = N/M; PLR = P/L; NLR = N/L; LMR = L/M; NLR = N/L; PLR = P/L; and MLR = M/L, where L, M, N, P represent the lymphocyte (10^9^/L), monocyte (10^9^/L), neutrophil (10^9^/L) and platelet (10^9^/L) counts, respectively, and continuous variables were converted to categorical variables. In this study, the best cut-off values for laboratory variables were calculated by using receiver operating characteristic (ROC) curve analysis. The cut-off values were as follows: ALB (43 g/L), C-reactive protein (CRP) (3.78 mg/L), PMR (518.0), NMR (7.0), PLR (206.0), NLR (3.36), LMR (7.25).

### 2.3. Treatment and Follow-Up

Patients in our study underwent modified radical mastectomy or breast conserving surgery. Most patients received standard postoperative adjuvant systemic treatment, such as chemotherapy, endocrine therapy, trastuzumab, or radiotherapy to the chest wall and local lymph nodes. Routine re-examination of routines every three months for the first two years, every six months for the next three years and annually thereafter was performed after surgery. The time from the date of surgery to the date of death or the final follow-up was defined as OS. The last follow-up was conducted in December 2020.

### 2.4. Statistical Analysis

The analysis was performed using SPSS 24.0 (IBM, Armonk, NY, USA) and R Software Version 4.0.2 (R Statistical Computing Foundation, Vienna, Austria). Differences in proportions of clinical characteristics were analyzed by the Chi-squared test. Univariate and multivariate Cox regression analysis were performed to find independent prognostic indicators of OS. We identified four optimal hematological indexes, ALB, NMR, NLR and LMR for the SIRS, which was constructed based on lowest Akaike information criterion (AIC) value. AIC is a standard to measure the fitness of statistical models based on the concept of entropy, which can balance the complexity of the estimated model with the quality of the data fitted by the model. Simply put, the model with lower AIC value has a higher simplicity and accuracy [17]. The SIRS of every patient was calculated according to the status of hematological indexes, which above the cut-off value was defined as 1, and below the cut-off value was defined as 0. Therefore, the computational formula of SIRS was as follows: SIRS = sum (each status of hematological index × corresponding regression coefficient). The patients were divided into low-SIRS and high-SIRS groups according to the median value of the SIRS. A nomogram was constructed by using ‘rms’ package of R software. The performance characteristics of the predictive nomogram was examined by calibration plots. The predictive accuracy of the nomogram for OS was evaluated by performing time-dependent receiver operating characteristic (ROC) curve analysis. Statistical significance was defined as *p*-value < 0.05, all *p* values were two-tailed.

## 3. Results

### 3.1. Patients’ Clinical Characteristics

1583 breast cancer patients were enrolled in this research from August 2012 to December 2015. Patients were randomly separated into a training (n = 1187) or external validation cohort (n = 396) in 3:1 ratio. The training cohort was used to generate the SIRS formula and prognostic model, and the external validation cohort was used to verify the accuracy of the prognostic model. There was no statistically significant difference in clinical characteristics between these two cohorts. The baseline clinical characteristics of the patients are summarized in Table 1. There were 1346 patients (85.1%) aged less than 60 years old; 558 patients (35.2%) accompanied by vascular cancer emboli; 47.7% patients with lymph node metastasis during surgery. Among them, there were 328 cases (20.7%) of Luminal A, 883 cases (55.8%) of Luminal B, 184 cases (11.6%) of HER2 positive and 188 cases (11.9%) of triple negative breast cancer (TNBC). 

### 3.2. The Construction of SIRS and Its Relationship with OS

**Figure 1:**
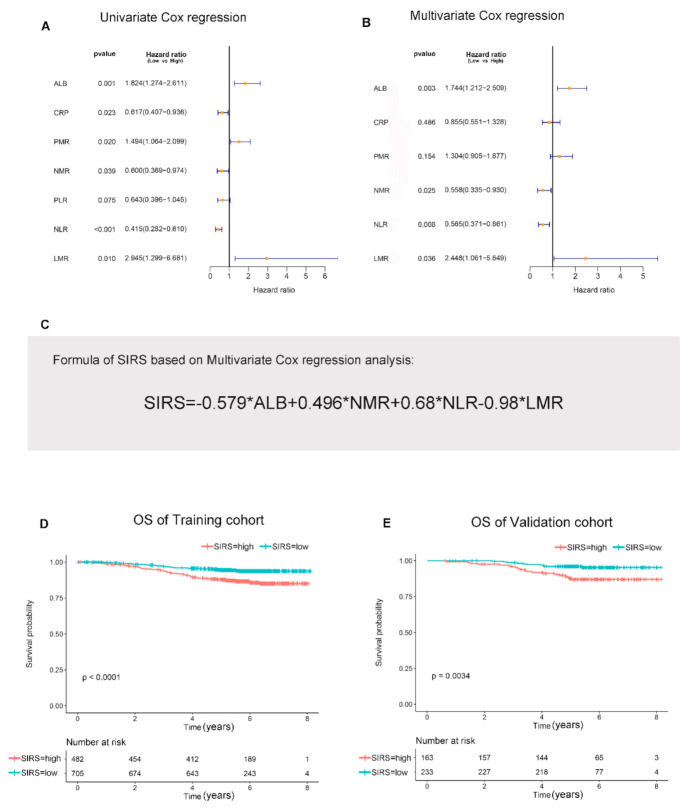
**SIRS is associated with OS in patients with breast cancer.** (**A**,**B**). Results of the univariate Cox regression analysis and multivariate Cox regression analysis between hematological markers of inflammation and overall survival in the training cohort. (**C**). The computational formula of SIRS. (**D**). Kaplan–Meier curves for the overall survival of patients in the high- and low-SIRS groups in the training cohort. (**E**). Kaplan–Meier curves for the overall survival of patients in the high- and low-SIRS groups in the validation cohort. **Abbreviation:** C-reactive protein (CRP), serum albumin (ALB), platelet-monocyte ratio (PMR), neutrophil-monocyte ratio (NMR), platelet-lymphocyte ratio (PLR), neutrophil-lymphocyte ratio (NLR), lymphocyte-monocyte ratio (LMR).

Univariate and multivariate Cox regression analysis were performed to find independent prognostic hematological factors of OS. The results showed that ALB, NMR, NLR and LMR were independent prognostic hematological factors for OS in breast cancer patients (Figure 1A,B). In order to simplify the calculation, hematological indexes of ALB, NMR, NLR and LMR which above the cut-off value was defined as 1, and below the cut-off value was defined as 0. Then, the SIRS was constructed based on the lowest AIC value, and the results showed that the lowest AIC value could be obtained only when ALB, NMR, NLR and LMR were all included in the model. The lack of any hematological indicators would increase the AIC value, that is, reduce the accuracy of the SIRS (Supplemental Table S1). According to the AIC results, the regression coefficients for each hematological index were calculated using multivariate Cox regression analysis (Supplemental Table S2). Eventually, the SIRS of every patient is calculated as SIRS = −0.579 × ALB + 0.496 × NMR + 0.68 × NLR − 0.98 × LMR (Figure 1C). On the basis of the median value of SIRS, the score of patients less than 0 were defined as low-SIRS group, and greater than 0 were defined as high-SIRS group. The result of Kaplan–Meier analysis showed that both in the training cohort and the external validation cohort, patients with high-SIRS had a significantly worse prognosis than patients with low-SIRS (Figure 1D,E).

### 3.3. The Association between SIRS and the Clinical Characteristics

**Figure 2:**
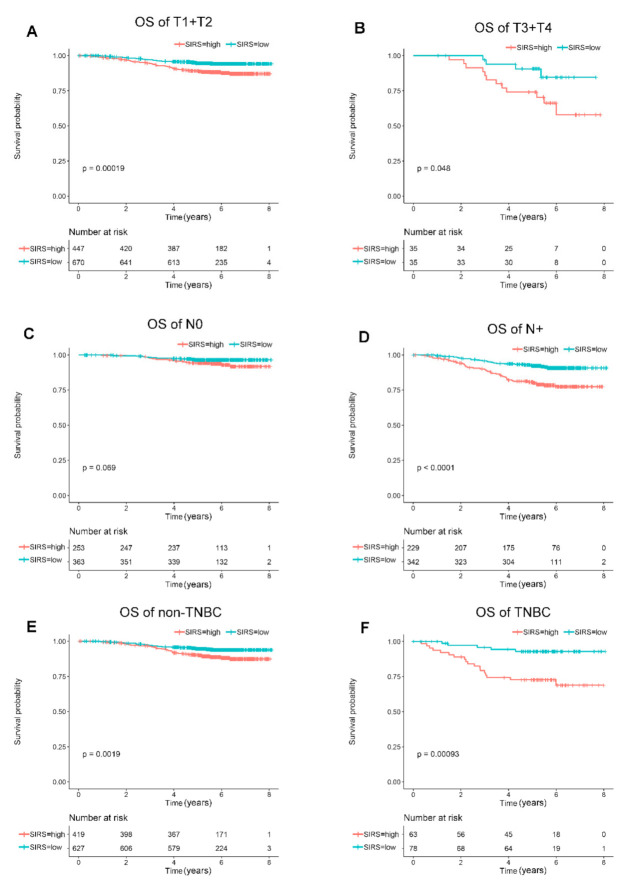
**Subgroup-based Kaplan–Meier analysis for breast cancer patients between SIRS and OS.** (**A**). Kaplan–Meier curves for the overall survival of patients with T1/T2 tumor. (**B**). Kaplan–Meier curves for the overall survival of patients with T3/T4 tumor. (**C**). Kaplan–Meier curves for the overall survival of patients with no lymph node metastasis. (**D**). Kaplan–Meier curves for the overall survival of patients with positive lymph node metastasis. (**E**). Kaplan–Meier curves for the overall survival of non-triple negative breast cancer patients. (**F**). Kaplan–Meier curves for the overall survival of triple negative breast cancer patients.

Then the relationship between SIRS and the clinical characteristics was explored. The result showed SIRS was significantly correlated with multifocality and N stage at the time of surgery. Patients with multiple lesions and advanced N stage showed a higher SIRS. However, age, histological grade, vascular cancer emboli (VCE), T stage and IHC subtype were not statistically correlated (Table 2). To analyze the role of SIRS in different breast cancer subgroups, a further subgroup analysis was conducted. In T1/2 or T3/4 breast cancer patients, there was a statistically significant difference in OS between low-SIRS and high-SIRS (Figure 2A,B). Meanwhile, among patients with lymph node metastasis breast cancer, the OS of high-SIRS patients was worse, while among patients without lymph node metastasis, no statistical difference was observed between the two groups (Figure 2C,D). As shown in Figure 2E,F, for both TNBC and non-TNBC, patients with high-SIRS had significantly worse overall survival than those with low-SIRS, and the difference in survival between low-SIRS and high-SIRS patients was more pronounced in patients with triple negative breast cancer.

### 3.4. SIRS Is an Independent Prognostic Factor for OS 

To determine whether the SIRS was an independent prognostic indicator for OS of breast cancer patients, univariate Cox regression analysis and multivariate Cox regression analysis were performed among the clinical characteristics and SIRS. In univariate analysis, age, SIRS, VCE, T stage, N stage and pathological classification were prognostic indicators for OS in breast cancer patients, while multiple lesions had no prognostic significance. Subsequently, significant indicators from univariate analysis were included in the multivariate analysis, and the multivariate analysis results showed that age, SIRS, VCE, T stage, N stage, and pathological classification were independent prognostic factors for breast cancer patients (Table 3). These results suggested that SIRS was still an independent prognostic factor in breast cancer patients after clinical features were included.

### 3.5. Construct and Verify a Nomogram Based on SIRS

**Figure 3:**
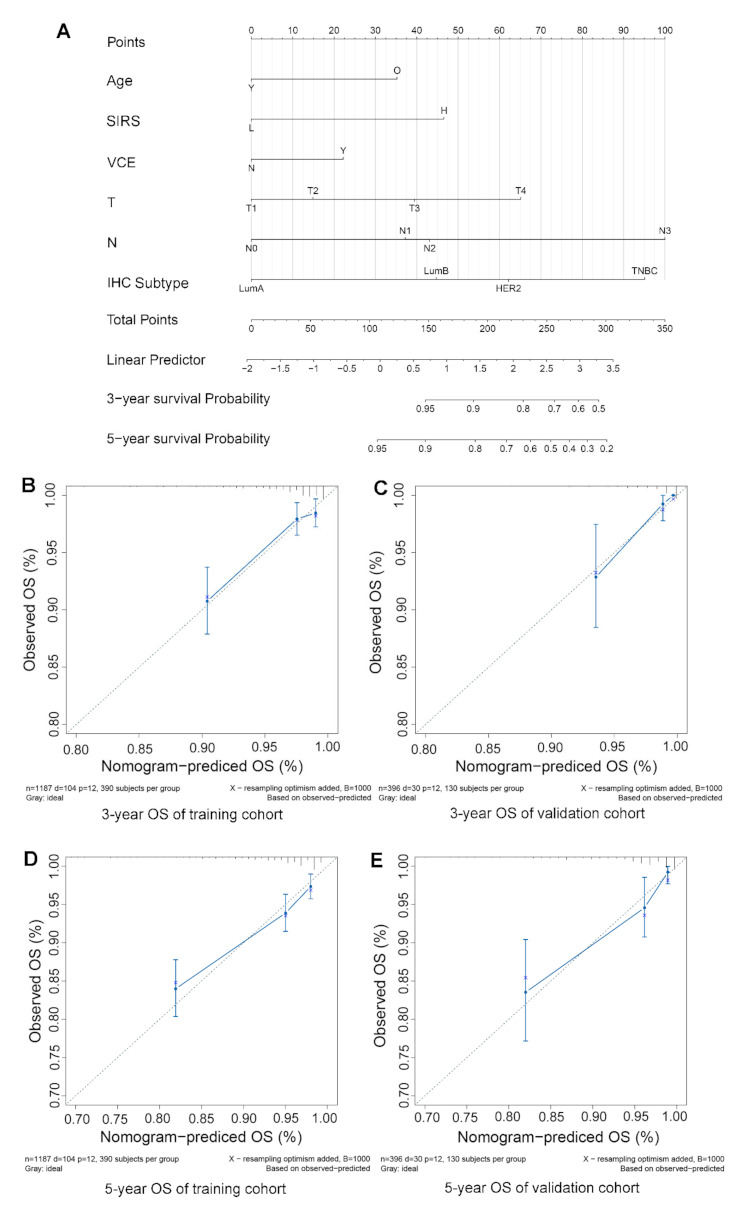
**Nomogram predicting the overall survival for patients with breast cancer.** (**A**). Nomogram for predicting OS of breast cancer patients after surgery. (**B**,**C**). Calibration plot of the nomogram for 3-year survival in the training cohort and external validation cohort. (**D**,**E**). Calibration plot of the nomogram for 5-year survival in the training cohort and external validation cohort. **Abbreviation:** vascular cancer emboli (VCE).

**Figure 4:**
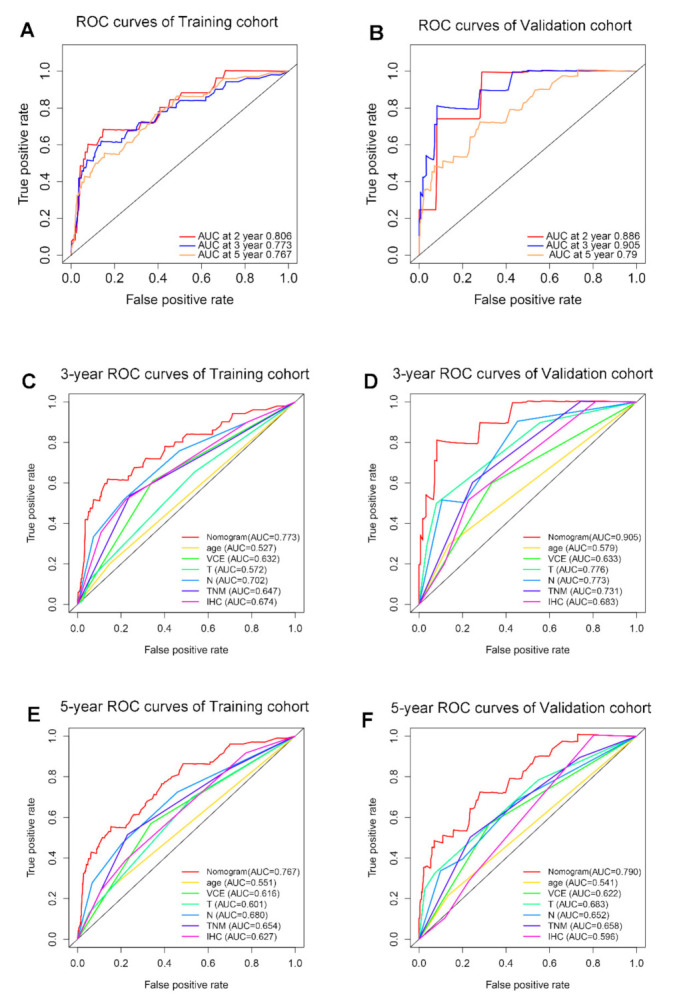
**The predictive performance of the nomogram is better than that of traditional prognostic factors.** (**A**). AUC of time-dependent ROC curves verified the prognostic accuracy of the nomogram in the training cohort. (**B**). AUC of time-dependent ROC curves verified the prognostic accuracy of the nomogram in the external validation cohort. (**C**,**D**). AUC of ROC curves compared the prognostic accuracy for 3-year survival of the nomogram and traditional prognostic factors in the training cohort and external validation cohort. (**E**,**F**). AUC of ROC curves compared the prognostic accuracy for 5-year survival of the nomogram and traditional prognostic factors in the training cohort and external validation cohort. **Abbreviation:** vascular cancer emboli (VCE).

To provide a cost-effective, rapid, stable, and flexible prediction model, independent prognostic indicators were used to construct a nomogram, including age, SIRS, VCE, T stage, N stage, and IHC classification (Figure 3A). In training or external validation cohort, the calibration plots showed high consistency between observation and prediction in predicting 3-year and 5-year survival (Figure 3B–E). Time-dependent ROC curve analysis was performed to evaluate the predictive accuracy of the nomogram for OS. When the nomogram was used to predict two-year, three-year and five-year survival rates, the area under the curve (AUC) in the training cohort was 0.806, 0.773 and 0.767, respectively; in the external validation cohort, it was 0.866, 0.905 and 0.79, respectively (Figure 4A,B). Then ROC curve analysis was used to compare the predictive ability between the nomogram and traditional clinical indicators, such as age, VCE, T stage, N stage, IHC subtype and TNM. The results showed that both in the training cohort and the external validation group, the AUC of nomogram is much higher than the traditional clinical indicators (Figure 4B–E), which meant that the nomogram had a higher accuracy in predicting survival than the traditional indicators. 

## 4. Discussion

In this research, we retrospectively collected hematological markers related to inflammatory response, clinicopathological features, and outcomes of 1583 stage I-III breast cancer patients undergoing modified radical mastectomy or breast conserving surgery. Neutrophils, monocyte, platelets, lymphocytes, and inflammatory markers such as CRP and ALB were included.

In the univariate and multivariate Cox regression analysis of OS, SIRS based on the status of ALB, LMR, NLR and NMR was an independent prognostic indicator. By combining hematological indicator and clinical features, we successfully established a model for predicting OS in breast cancer patients and the predictive indicators used were simple and easy to obtain. 

Chronic inflammation is related to the occurrence, progression, and prognosis of tumors [18]. Meanwhile, inflammatory cells are an important part of tumor microenvironment (TME) and mediate TME. In addition, systemic inflammation is closed related with the local inflammatory response [19]. Growing evidence suggests that local and systemic inflammatory responses affect survival in patients with cancer, in which the loss of P53 played a role in neutrophils promoting breast cancer metastasis [20,21].

Serum albumin, as a routine item in patients’ biochemical tests, reflects patients’ nutritional levels, which are also associated with chronic inflammatory responses. Low levels of serum albumin can occur in patients with malnutrition and chronic infection. At the same time, patients with low levels of ALB have worse prognoses. Low levels of serum ALB are considered as an independent adverse prognostic indicator [14,15]. Lymphocytes play a role in the specific immune response, in which CD8+T cells are the main effector cells that kill tumors [22]. Macrophages contribute to the growth and escape of tumors by secreting cytokines. Monocytes can further differentiate into macrophages when they enter the tissue, which helps the progression of the tumor [23]. It makes sense in theory that elevated LMR has been associated with better outcomes for many cancers, including breast cancer [24].

Neutrophils promote metastasis of tumor cells and are associated with patient prognosis, in which neutrophil extracellular traps (Nets) play an important role [25]. Elevated NLR indicates a relative decrease in lymphocytes or increase in neutrophils, which alters the TME and causes tumor progression, leading to a poor prognosis in cancer patients [16]. NMR, as an inflammatory marker, has rarely been reported in tumor prognosis studies. Sun et al. reported that elevated NMR was associated with poor prognosis in patients with gastric cancer, but it failed to be an independent prognostic factor [26]. In this study, elevated NMR was an independent adverse prognostic factor for breast cancer patients, suggesting that neutrophils may play a greater role in promoting the development of breast cancer than macrophages. 

Compared with previous prognostic models, the accuracy of nomogram model based on SIRS was higher than that of TNM stage. At the same time, this nomogram is more operable, economical, and suitable for widespread application in primary hospital than 21-gene, MammaPrint, or PAM50. The 21-gene testing has been proved to predict the prognosis of hormone receptor-positive and HER2-negative early breast cancer patients. But its role in the prediction of other molecular subtypes of breast cancer has not been proved [27], and the 21-gene testing is uneconomical. Compared with 21-gene testing, our prognostic model is more broadly applicable and less expensive. PAM50 is used to define breast cancer intrinsic subtypes and recurrence risks based on mRNA levels of 50 genes, and it has been reported that the C-index of PAM50 in predicting survival of breast cancer was 0.63–0.67 [28,29], indicating that PAM50 is not better than our model in predicting survival. MammaPrint is the first prospectively validated genomic prognostic model analysis for breast cancer. A total of 70 genes were included in the model with the C-index of 0.614 [30]. Compared with MammaPrint, our prognostic model is more precise and economical. Among the prognostic models that included blood markers, Cho et al. conducted a small sample study and only verified the role of PLR, but failed to include important factors that have been confirmed, such as LMR, NLR, NMR and ALB [31]. Huang et al. reported that the systemic inflammation score (SIS) based on ALB and LMR could predict the survival of breast cancer patients [32]. Zheng et al. found that fibrinogen-albumin ratio and platelet-lymphocyte ratio score was a prognostic factor of breast cancer [33], and NLR has been shown to predict the efficacy of neoadjuvant chemotherapy in breast cancer [34,35]. However, these indicators all partially analyzed the influence of systemic inflammation on breast cancer. Comparatively, SIRS included more systemic inflammation indicators and was shown to be a comprehensive indicator of systemic inflammation. Systemic immune-inflammation index (SII) was also reported as a prognostic indicator of breast cancer, which was calculated by neutrophil, platelet and lymphocyte levels, and the AUC of SII based on ROC analysis was 0.625 [36]. Compared with SII, the SIRS included more inflammatory indicators and was more comprehensive, and our model based on SIRS had more accurate predictive ability to predict survival of breast cancer patients. 

Systemic inflammation has been proved to be associated with the prognosis of different cancers. For example, neutrophil-to-lymphocyte ratio (NLR) was associated with the outcomes of esophageal carcinoma [37], and SII could predict the survival of non-small cell lung cancer patients [38], cervical cancer patients [39] and colorectal cancer patients [40]. SIRS is a more comprehensive systemic inflammatory marker, including more hematological indicators than previous markers of systemic inflammation. Therefore, it is reasonable to infer that SIRS can also be used to predict the prognosis of other cancers, such as lung cancer, colorectal cancer, esophageal cancer and so on. SIRS therefore has potential to help clinicians to effectively predict the prognosis of different cancer patients and to individualize treatment.

To our knowledge, this prediction model, considering the importance of inflammation in cancer prognosis, has enrolled the largest sample study to date and included molecular hematological indicators that are more comprehensive, including ALB, CRP, PMR, NMR, PLR, NLR and LMR. Meanwhile, we classified four systemic inflammatory indicators and scored each patient according to their weight in the prognosis of breast cancer patients, which was named SIRS. Multifocality and advanced N stage patients had higher SIRS, however the reason is not clear at present, whether patients with high-SIRS progress faster, or if patients’ immune status changes with the progression of the disease, resulting in an increase in SIRS, something which needs to be confirmed by further studies.

This study suggests that SIRS can provide additional prognostic information for traditional clinicopathologic features in terms of host immune status. These results may help clinicians to screen patients with a potentially poor prognosis, adopt more aggressive treatment regimens, enhance postoperative follow-up, and perhaps help better understand the relationship between immunity, inflammation, and cancer prognosis. However, there are some limitations in the study. This study is a single-center retrospective study, which needs to be confirmed by multi-center prospective studies. SIRS is based on preoperative examinations of patients with operable breast cancer. Its applicability in patients with advanced, inoperable breast cancer has not yet been proven, and clinical data from patients with advanced breast cancer is needed to explore the predictive ability of SIRS in inoperable breast cancer patients.

In conclusion, SIRS is an independent prognostic factor for the prognosis of operable breast cancer patients, which is easy to obtain and stable. Nomograms based on SIRS can accurately predict the prognosis of breast cancer patients.

## Figures and Tables

**Table 1:** Patient and tumor characteristics in the training cohort and external validation cohort.

Variables	Total (n = 1583)		Training Cohort (n = 1187)		Validation Cohort (n = 396)		*p*-Value
	**NO.**	**%**	**NO.**	**%**	**NO.**	**%**	
**Age**							0.621
≤60	1347	85.1	1007	84.8	340	85.9	
>60	236	14.9	180	15.2	56	14.1	
**Multifocality**							0.641
Yes	39	2.5	28	2.4	11	2.8	
No	1544	97.5	1159	97.6	385	97.2	
**Grade**							0.06
I	108	6.8	90	7.6	18	4.5	
II	903	57.0	681	57.4	222	56.1	
III	572	36.1	416	35.0	156	39.4	
**VCE**							0.577
Yes	558	35.2	423	35.6	135	34.1	
No	1025	64.8	764	64.4	261	65.9	
**T stage**							0.148
T1	717	45.3	545	45.9	172	43.4	
T2	760	48.0	572	48.2	188	47.5	
T3	60	3.8	41	3.5	19	4.8	
T4	46	2.9	29	2.4	17	4.3	
**N stage**							0.053
N0	828	52.3	616	51.9	212	53.6	
N1	402	25.4	303	25.5	99	25.0	
N2	207	13.1	168	14.2	39	9.8	
N3	146	9.2	100	8.4	46	11.6	
**IHC subtype**							0.520
Luminal A	328	20.7	256	21.6	72	18.2	
Luminal B	883	55.8	652	54.9	231	58.3	
HER2+	184	11.6	138	11.6	46	11.6	
TNBC	188	11.9	141	11.9	47	11.9	
**ALB (g/L)**							0.783
<43	830	52.4	620	52.2	210	53.0	
≥43	753	47.6	567	47.8	186	47.0	
**CRP (mg/L)**							0.361
<3.78	1361	86.0	1026	86.4	335	84.6	
≥3.78	222	14.0	161	13.6	61	15.4	
**PMR**							0.704
<518	587	37.1	437	36.8	150	37.9	
≥518	996	62.9	750	63.2	246	62.1	
**NMR**							0.720
<7.0	346	21.9	262	22.1	84	21.2	
≥7.0	1237	78.1	925	77.9	312	78.8	
**PLR**							0.965
<206	1424	90.0	1068	90.0	356	89.9	
≥206	159	10.0	119	10.0	40	10.1	
**NLR**							0.936
<3.36	1377	87.0	1033	87.0	344	86.9	
≥3.36	206	13.0	154	13.0	52	13.1	
**LMR**							0.410
<7.25	1401	88.5	1046	88.1	355	89.6	
≥7.25	182	11.5	141	11.9	41	10.4	

**Abbreviation:** vascular cancer emboli (VCE), C-reactive protein (CRP), serum albumin (ALB), platelet-monocyte ratio (PMR), neutrophil-monocyte ratio (NMR), platelet-lymphocyte ratio (PLR), neutrophil-lymphocyte ratio (NLR), lymphocyte-monocyte ratio (LMR).

**Table 2:** Relationship between SIRS and clinical characteristics in training cohort.

Variables	Total (n = 1187)		SIRS High (n = 482)		SIRS Low (n = 705)		*p*-Value
	**NO.**	**%**	**NO.**	**%**	**NO.**	**%**	
**Age**							0.072
≤60	1007	84.8	398	82.6	609	86.4	
>60	180	15.2	84	17.4	96	13.6	
**Multifocality**							0.004
Yes	28	2.4	4	0.8	24	3.4	
No	1159	97.6	478	99.2	681	96.6	
**Grade**							0.588
I	90	7.6	40	8.3	50	7.1	
II	681	57.4	269	55.8	412	58.4	
III	416	35.0	173	35.9	243	34.5	
**VCE**							0.477
Yes	423	35.6	166	34.4	257	36.5	
No	764	64.4	316	65.6	448	63.5	
**T stage**							0.187
T1	545	45.9	212	44.0	333	47.2	
T2	572	48.2	235	48.8	337	47.8	
T3	41	3.5	23	4.8	18	2.6	
T4	29	2.4	12	2.5	17	2.4	
**N stage**							0.017
N0	616	51.9	253	52.5	363	51.5	
N1	303	25.5	105	21.8	198	28.1	
N2	168	14.2	72	14.9	96	13.6	
N3	100	8.4	52	10.8	48	6.8	
**IHC subtype**							0.096
Luminal A	256	21.6	105	21.8	151	21.4	
Luminal B	652	54.9	271	56.2	381	54.0	
HER2+	138	11.6	43	8.9	95	13.5	
TNBC	141	11.9	63	13.1	78	11.1	

**Abbreviation:** vascular cancer emboli (VCE).

**Table 3:** Results of the univariate Cox regression analysis and multivariate Cox regression analysis for OS among the clinical characteristics and SIRS.

Variables	Univariate Cox Analysis		Multivariate Cox Analysis	
	**HR (95% CI)**	***p*-Value**	**HR (95% CI)**	***p*-Value**
**Age**		0.003		0.014
≤60	Reference		Reference	
>60	1.965(1.267–3.046)		1.778(1.124–2.814)	
**SIRS**		<0.001		<0.001
Low	Reference		Reference	
High	1.543(1.266–1.879)		2.399(1.607–3.583)	
**Multifocality**		0.340		
No	Reference			
Yes	0.383(0.053–2.747)			
**Grade**		0.027		0.433
I	Reference		Reference	
II	0.906(0.410–2.000)	0.807	0.591(0.259–1.351)	0.213
III	1.553(0.703–3.433)	0.276	0.678(0.294–1.566)	0.363
**VCE**		<0.001		0.039
No	Reference		Reference	
Yes	2.426(1.647–3.573)		1.566(1.022–2.398)	
**T stage**		<0.001		0.021
T1	Reference		Reference	
T2	1.995(1.279–3.112)	0.002	1.395(0.879–2.211)	0.158
T3	4.538(2.147–9.588)	<0.001	2.210(0.992–4.924)	0.052
T4	4.947(2.166–11.298)	<0.001	3.428(1.455–8.076)	0.005
**N stage**		<0.001		<0.001
N0	Reference		Reference	
N1	1.887(1.111–3.204)	0.019	2.074(1.194–3.603)	0.010
N2	2.560(1.436–4.566)	0.001	2.137(1.138–4.014)	0.018
N3	8.140(4.883–13.572)	<0.001	5.876(3.341–10.334)	<0.001
**IHC subtype**		<0.001		0.001
Luminal A	0.183(0.085–0.395)	<0.001	0.208(0.093–0.466)	<0.001
Luminal B	0.481(0.297–0.779)	0.003	0.421(0.253–0.701)	0.001
HER2+	0.568(0.293–1.105)	0.096	0.565(0.287–1.113)	0.099
TNBC	Reference		Reference	

**Abbreviation:** vascular cancer emboli (VCE).

## Data Availability

The data presented in this study are available on request from the corresponding author.

## Supplementary Materials

The following are available online at https://www.mdpi.com/article/10.3390/jpm11050413/s1, Table S1: Akaike information criterion (AIC) for the prognostic model, Table S2: Regression coefficient of each model based on multivariate Cox regression analysis.
