# Supplementary material for: A Systemic Inflammation Response Score for Prognostic Prediction of Breast Cancer Patients Undergoing Surgery
Source: J Pers Med. 2021 May 14;11(5):413. doi: 10.3390/jpm11050413 (PMC8156296; doi:10.3390/jpm11050413)
Supplement: Supplementary file 1 [file jpm-11-00413-s001.zip › jpm-1197525-supplementary.pdf]

Supplemental Table S1. Akaike information criterion (AIC) for the prognostic model.

Model	Prognostic signature combination	AIC
1	ALB + NMR + NLR + LMR	1908.2
2	ALB + NLR + LMR	1910.5
3	ALB + NMR + NLR	1913.5
4	ALB + NMR + LMR	1916.5
5	NMR + NLR + LMR	1916.7

Supplemental Table S2. Regression coefficient of each model based on multivariate Cox regression analysis.

Prognostic factors	Regression coefficient based on multivariate Cox regression				
	Model 1	Model 2	Model 3	Model 4	Model 5
ALB	-0.579	-0.555	-0.593	-0.607	NA
NMR	0.496	NA	0.422	0.631	0.447
NLR	0.680	0.775	0.773	NA	0.718
LMR	-0.980	-0.901	NA	-1.119	-0.101
